# Short rotation plantations policy history in Europe: lessons from the past and recommendations for the future

**DOI:** 10.1002/fes3.86

**Published:** 2016-08-19

**Authors:** Kevin N. Lindegaard, Paul W. R. Adams, Martin Holley, Annette Lamley, Annika Henriksson, Stig Larsson, Hans‐Georg von Engelbrechten, Gonzalo Esteban Lopez, Marcin Pisarek

**Affiliations:** ^1^ Crops for Energy Ltd15 Sylvia AvenueKnowleBristolBS3 5BXUK; ^2^ Department of Mechanical EngineeringFaculty of Engineering and DesignInstitute for Sustainable Energy and Environment (I∙SEE)University of BathClaverton DownBathBA2 7AYUK; ^3^ Centre for Sustainable Energy3 St Peter's Court, Bedminster ParadeBristolBS3 4AQUK; ^4^ Henriksson Salix ABAlmhög241 92EslövSweden; ^5^ EWBSpannmålsgatan 28SE‐268 32SVALÖVSweden; ^6^ Agraligna GmbHOststrasse 738315Schladen/OT BeuchteGermany; ^7^ Agencia Provincial de la Energía de GranadaEdificio CIE ‐ 1ºPlanta. Avda. Andalucía s/n.18015GranadaSpain; ^8^ PGNiG TERMIKA SASiedziba główna ‐ Elektrociepłownia Żerań, ul. Modlińska 1503‐216WarszawaPoland

**Keywords:** Biomass, energy crop, policy, short rotation coppice, short rotation plantations

## Abstract

Short rotation plantations (SRPs) are fast‐growing trees (such as willow (*Salix* spp.), poplar (*Populus* spp.) and *Eucalyptus*) grown closely together and harvested in periods of 2–20 years. There are around 50,000 hectares of SRPs in Europe, a relatively small area considering that there have been supportive policy measures in many countries for 30 years. This paper looks at the effect that the policy measures used in different EU countries have had, and how other external factors have impacted on the development of the industry. Rokwood was a 3‐year European funded project which attempted to understand the obstacles and barriers facing the woody energy crops sector using well established methods of SWOT and PESTLE analysis. Stakeholder groups were formed in six different European regions to analyze the market drivers and barriers for SRP and propose ways that the industry could make progress through targeted research and development and an improved policy framework. Based upon the outcomes of the SWOT and PESTLE analysis, each region produced a series of recommendations for policymakers, public authorities, and government agencies to support the development, production, and use of SRP‐derived wood fuel in each of the partner countries. This study provides details of the SRP policy analysis and reveals that each region shared a number of similarities with broad themes emerging. There is a need to educate farmers and policymakers about the multifunctional benefits of SRPs. Greater financial support from regional and/or national government is required in order to grow the SRP market. Introducing targeted subsidies as an incentive for growers could address lack of local supply chains. Long‐term policy initiatives should be developed while increasing clarity within Government departments. Research funding should enable closer working between universities and industry with positive research findings developed into supportive policy measures.

## Introduction

Short rotation plantations (SRPs) offer an opportunity to increase energy security by providing a local source of low carbon renewable biomass fuel. Bioenergy offers an alternative to fossil fuels, reductions in greenhouse gas emissions, and assists in the economic development of rural communities (Defra, 2007). Policies have therefore been implemented across Europe to promote bioenergy and the domestic planting of perennial energy crops (Mangan 1997; Thornley and Cooper 2008; EC 2009; Adams 2011; Natural England, 2013; Adams & Lindegaard, 2016). Despite the various policy instruments, grants, and incentives implemented, the cultivation of woody energy crops has proceeded at a low rate across the EU (IEE 2009; Aebiom 2015; EurObserv'ER, 2015). The research is part of an EU‐funded project that is exploring ways to increase the cultivation of SRPs throughout Europe; hence, the hypothesis is that the positive benefits of SRP outweigh potential negative impacts. This paper provides an overview of the historical development of SRPs in Europe drawing on specific policy and market examples from six EU countries. An assessment is conducted of the current state of play for the SRP market in each country, with an analysis performed to provide policy recommendations for the future development of SRPs in Europe.

### History of SRPs in Europe

Short rotation plantations (SRPs) are fast‐growing trees grown closely together and harvested in periods of 2–20 years. Trees that are cultivated this way include willow (*Salix* spp.), poplar (*Populus* spp.), *Eucalyptus*, and *Robinia* (Rokwood, 2015a). SRPs have been considered as an option in modern agriculture for biomass energy and fiber production for over 40 years, although the historical use can be traced back to centuries (Bergendorff and Emanuelsson 1996). Initially, interest was sparked in the early 1970s by the potential shortage in pulp wood used for paper and cardboard production (Anon, 1980; Richards 1987). This potential land use also received significant attention in the wake of the OPEC oil crisis of 1973, with the oil embargo increasing oil prices and leading to supply shortages (Ross 2013). Countries like Sweden and the Netherlands with low levels of indigenous fossil fuels were particularly exposed to this issue and endured energy rationing (Chitadze 2012; Verwijst et al. 2013). In light of this incident, the need for greater security of energy supply became important and research on willow for biomass energy began in Sweden and United Kingdom (Dawson 1992; Mangan 1997; Lindegaard et al. 2001).

Initial research efforts suggested that high yields could be achieved on marginal land and an industry started to develop. The first commercial willow plantings took place in Sweden in 1981 and cuttings suppliers were offering large volumes of material from 1985; the first mechanized planter (the Step Planter) was developed in 1986 and Svalöf‐Weibull AB began commercial willow breeding in 1987 (Larsson 1998; Verwijst et al. 2013).

The industry began to grow with the introduction of set‐aside in 1988 under the Common Agricultural Policy (CAP) (EC 1988). This program imposed production quotas and forced farmers to take a proportion of their land out of food production in order to control the oversupply of agricultural commodities such as milk and grain. There were suggestions at the time that 6 million hectares of UK farmland would need to be removed from food production; SRPs therefore emerged as an attractive diversification option (Dawson 1992).

Other geo‐political factors also stimulated the industry. The realization that over reliance on fossil fuels was causing global warming, led to the Earth Summit in Rio in 1992 and the signing of Kyoto Protocol in 1997 (UNCED, 1992; Keating 1993; UNFCCC, 1997). The need for large‐scale carbon emissions reduction led some countries like Sweden and Denmark to adopt carbon taxes (McCormick and Kåberger 2005). This gave a favorable advantage to renewable energy and home grown biomass production (ETSU, 1999; Mola‐Yudego and Pelkonen 2008).

Throughout the history of the SRP sector there have been key breakthroughs in research and technology development (Verwijst et al. 2013). For instance, there are numerous breeding and selection programs for SRPs in Sweden, UK, Italy, Belgium, Germany, Poland, and Spain (Lindegaard and Barker 1997; Larsson 1998; Karp et al. 2011; Isebrands and Richardson 2014). From these efforts there are some exceptional, high‐ yielding and disease‐resistant varieties (Danfors et al. 1997; Lindegaard et al. 2001, 2011; Caslin et al. 2012). In addition, planting and harvesting technology has been developed, making it easier to ensure a good establishment and efficient harvesting (PAMI, 2003; Spinelli et al. 2008, 2009; Schweier and Becker 2012; Henriksson and Henriksson 2015).

Nonetheless, despite this promising start and 30 years of supportive policy measures in many countries, the industry has faltered and there are currently estimated to be 50,000 hectares of SRPs in the EU28 (Aebiom 2015). In many countries, there has been a similar trend with relatively large areas of SRPs being established in a short time followed by a rapid decline (See section Short Rotation Plantations (SRP) policy review).

### Rokwood

Rokwood was a six‐country study which aimed to make the regionally based production of woody biomass economically attractive, technically feasible, and environmentally sustainable (Rokwood, 2015b). SRPs provide a quick and efficient way of producing large volumes of woody biomass where there is a local market or need. Besides their high productivity, SRPs offer further advantages such as providing landscape diversity, increased biodiversity compared to annual crops, and numerous ecosystem services such as reduction in soil erosion, reductions in nutrient leaching, and a possible approach to flood mitigation (Johnston et al. 2015; Adams and Lindegaard 2016; Styles et al. 2016). These promising attributes are not being fully exploited due to a variety of obstacles and barriers hindering or even preventing the further development of the SRP sector (Adams et al. 2011; Lindegaard 2013a,b). These obstacles and barriers comprise missing or unfavorable legal framework conditions, missing financial support, and various technical and nontechnical barriers.

Rokwood as a trans‐European research project attempted to confront these issues to find innovative ways to increase the market penetration of woody energy crops. The project involved a large consortia of 20 partners from six European regions (Northern Germany, South West England, Mazovia in Poland, Skåne in Sweden, Andalusia in Spain, and the Midlands and Western Region of Ireland) as well as EUBIA, the European Biomass Industries Association. Each region is represented by three partners, respecting the triple‐helix concept (a business entity, a research entity, and a local or a regional authority) (Lindegaard et al. 2015). The six regions, in spite of their structural differences and levels of SRP engagement, face similar challenges in terms of developing the SRP market. Rokwood was intended to enforce the cooperation between these countries through a collective Joint Action Plan (JAP) for tackling the most important obstacles and barriers on the European level. By connecting these regions, Rokwood has striven to promote the exchange of established best practices and thus improve the economic growth of SRPs (Rokwood, 2015c).

### Aims and objectives

The first aim of this study is to briefly review the past history of SRP policy development in Europe and critique the main policies and incentives that were designed and implemented to increase SRP supply. The focus of the policy review is on those policies that specifically incentivized either production or use of SRP, or are beneficial to the farmed environment. The secondary aim of the research is to develop policy recommendations for each country, comparing and contrasting key themes and differences between countries. Policy recommendations are intended for policy makers, public authorities, and governmental agencies to support the development, production, and use of SRP‐derived wood fuel in each of the partner countries and beyond. Specific objectives required to address this aim include:

- Review key policies that have supported the development of SRP in Germany, Ireland, Poland, Spain, Sweden, and UK;
- Perform a SWOT (Strengths, Weaknesses, Opportunities, and Threats) and PESTLE (Political, Economic, Social, Technological, Legal, and Environmental) analysis to identify factors influencing the market for SRP in each of the six countries;
- Present the SRP policy recommendations produced as part of the Rokwood project.

## Methodology

**Figure 1:**
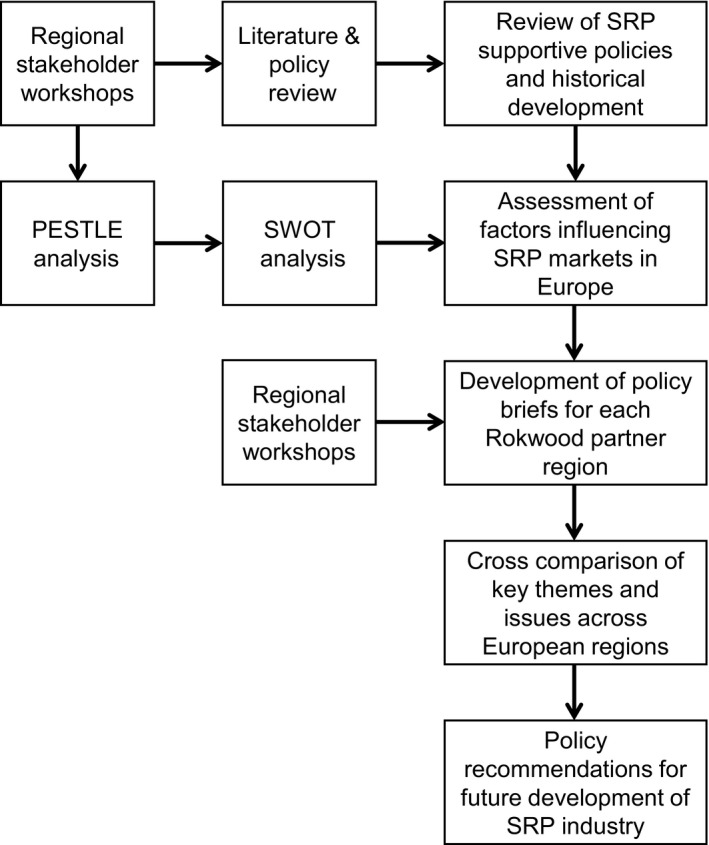
Overview of key stages of the research conducted for this paper.

This section describes the research methods that were followed to critique and assess different policies implemented to incentivize the cultivation of SRPs, analyze the factors influencing SRP markets, and present policy recommendations. Figure 1 provides a visual summary of the research methodology, and the following subsections describe the main research stages.

### Review of the key SRP policies for each country

The research commenced with a review of the policy measures that have been used in the past in Germany, Ireland, Poland, Spain, Sweden, and UK and the effect these had on the uptake of SRP. Consideration has also been given as to how other external factors have impacted on the development of the industry. For each country considered, a literature review was performed to identify policies that promoted the planting of SRPs, the use of SRPs or are beneficial to the farmed environment. The review focused on national and local policies and strategies, industry and research publications, and other literature in the public domain. Through project workshops, these policies were further identified and assessed for their relevance to the development of the SRP industry. Workshops consisted of varied stakeholders in each region with a knowledge and interest in SRP to provide different perspectives from policy, industry, agriculture, and research. A workshop was held in each region with the details of attendees provided in the Rokwood publications. The policies described in section Short Rotation Plantations (SRP) policy review provide a summary of some key policy examples in each partner country but do not provide an exhaustive list of all the policies identified.

### PESTLE and SWOT analysis

The Rokwood partners performed PESTLE (Political, Economic, Social, Technological, Legal, and Environmental factors) and SWOT (Strengths, Weaknesses, Opportunities, and Threats) analyses to identify all the factors that influence the SRP sector within their countries in order to prioritize and select those which could be best targeted by policymakers to help the industry expand.

The PESTLE analysis was performed first and represents a checklist of factors currently affecting the production and use of SRP, and those which are likely to affect it in the future (CPID, 2015a). It gives an overview of the whole environment from many different perspectives that need to be considered in policy development. Key questions that were considered for the PESTLE analysis were:

- What is the political situation of the country and how can it affect the SRP industry?
- What are the prevalent economic factors and market conditions for SRP?
- How much importance do social aspects have in the market for SRP?
- What technological innovations could arise that may affect the market structure?
- Which current legislations impact on the SRP industry and what influence can stakeholders have on future development of policy?
- What are the environmental considerations for the SRP industry?

The PESTLE analysis was undertaken by each cluster focusing on their corresponding region and developed in workshops (Parra‐López et al. 2015). These regional workshops consisted of different actors in the SRP market and included academics, agronomists, farmers, funders, plant breeders, and policymakers who have a specialist knowledge or influence on the development of SRP. To recruit workshop participants the Rokwood partners consulted expert knowledge outside the cluster group to list and rank external PESTLE factors. The method used involved key actors identified as:

- Experts in their field
- Able to look beyond the borders of their specialism
- Well‐renowned in the cluster for their ideas and opinion

Experts identified in the first step of the analysis were used. The key actors were not limited to persons active, in the current situation, within the SRP sector. Key actors may also be persons with prior experience from the SRP sector, representatives of organizations planning to expand within this sector, and other biomass fuel actors that as of today for different reasons refrain to involve in the SRP sector (e.g. operators of biomass plants not using SRP today). The workshop participants totaled 4–23 stakeholders in each region to ensure a good coverage of specialisms while still allowing active participation. The delegate's names remain anonymous as agreed at the onset of the research. Further details are available from the Rokwood representatives for each region. The Spanish cluster was the only project members to formally publish the PESTLE results (Sayadi et al. 2014; Parra‐López et al. 2015).

The first objective of the PESTLE analysis was to identify impediments and factors of success of regional regions through the evaluation of current markets and ascertain the barriers to growing SRP. A second objective was to identify potential policy mechanisms which stimulate growers and end users to develop the industry (Rokwood, 2014a).

The follow‐up SWOT analysis drew on the PESTLE outputs. A SWOT analysis is an established method of evaluating a situation or a market in a structured appraisal to help plan for the future (CPID, 2015b). In this context, it helps to identify strengths and weaknesses of the SRP market and map these to external opportunities and threats so that the most effective policies can be formulated to achieve market success. Each region populated a SWOT chart made up of four quadrants to identify “internal” strengths and weaknesses within the SRP industry alongside external opportunities and threats (Rokwood, 2014a). The most important factors in each quadrant (up to a maximum of 10) were then recorded. Factors that could make up a “common” SWOT across all the regions were then decided at a consortium meeting workshop. Results from the PESTLE and SWOT analyses are presented in section Summary outcomes of the PESTLE and SWOT analysis with further analysis provided in section Policy recommendations.

### Policy recommendations

**Figure 2:**
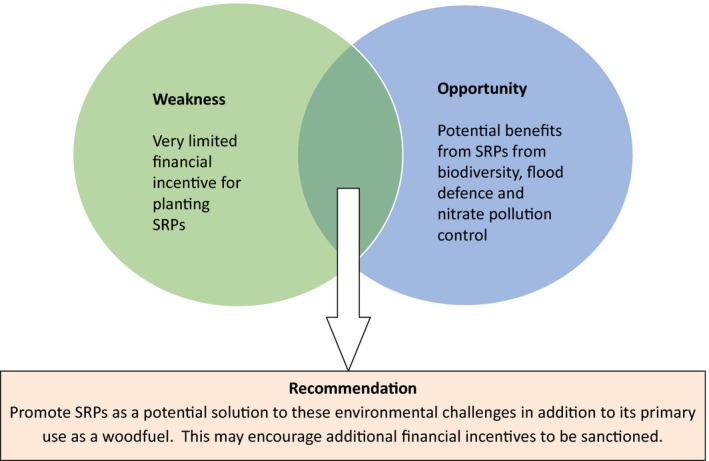
Example of using identified opportunities to negate weaknesses to SRPs in the UK.

**Figure 3:**
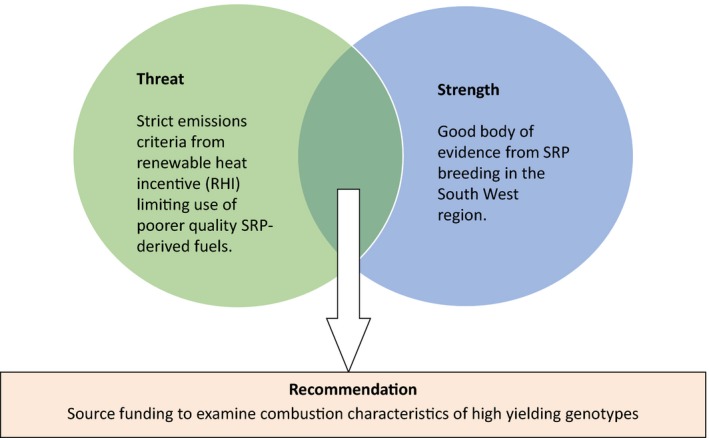
Example of using identified strengths to overcome threats to SRPs in the UK.

Findings from the policy and market review were consolidated and summarized to ensure all opportunities for and threats against SRP development were fully considered. This included an evaluation of the SWOT and PESTLE analyses as well as a detailed appraisal of the policy outcomes identified for each region (see section Policy recommendations). The SWOT analysis, in particular, was heavily used both to identify issues and to find potential solutions, with most of the issues being derived from weaknesses and threats in the SWOT analysis. Solutions were sought by looking for relationships, for example, a strength that could overcome a threat or an opportunity that could negate a weakness. Examples from the UK region are shown in Figs 2 and 3.

#### Stakeholder consultation exercise to validate policy measures

In order to validate the policy recommendations, a stakeholder consultation exercise was performed. To ensure that the final policy brief documents were suitably targeted and that they thoroughly addressed the key barriers identified, each of the Rokwood regions distributed draft copies of their briefs to relevant stakeholders for comment and peer review. These stakeholders included policymakers at local, regional, and national levels, academics specializing in related subjects (primarily agricultural, economic development, or energy‐based), and bioenergy consultants. The consultation exercise was an important final stage of the methodology to ensure the accuracy, appropriateness, validity, and completeness of the policy recommendations.

#### Policy briefs development for each of the six regions

Following the consultation exercise, each of the six regions produced a “policy brief” that drew upon the robust evidence base gathered from the stages described above and the outputs of the various Rokwood work packages (Rokwood, 2014b, 2015d,e,f,g,h). Due to the regional focus of the Rokwood project, the briefs were inevitably shaped by the characteristics of each region and are therefore primarily focused on influencing regional policy, although this does vary to some extent based on the structure of governance in each country. Once the policy recommendations were agreed following consultation, each region chose a number of recommendations to elaborate on more fully in the final policy briefs. Each of these draft recommendations was presented in line with the following four headings:

- What is the problem or issue?
- How could the problem or issue be addressed?
- Which stakeholders should take this forward?
- What are the benefits and potential outcomes?

Section Policy recommendations presents the key aspects of the policy briefs by assessing the policy mechanisms required to develop the SRP industry in the different European countries. A discussion of the main issues and solutions is presented within the policy recommendations to identify key themes and differences between regions.

## Short Rotation Plantations (SRP) Policy Review

This section provides a high‐level summary of the key policies and market situation for SRPs in each of the six European regions considered. Table 1 provides a summary of the different characteristics of each region, and the following subsections describe each region.

**Table 1:** Summary information regarding the six Rokwood regions (Lindegaard et al. 2015)

	Northern Germany	Midlands & Western Ireland	Mazovia, Poland	Andalusia, Spain	Skåne, Sweden	South West England
Population (millions)	19.5	1.1	5.3	8.4	1.3	5.3
Area (m ha)	13.77	3.25	3.56	8.76	1.09	2.38
Area of SRPs today (ha)	3600	117	1100	150–170	2042	93
Forest cover (m ha)	2.37	0.34	0.85	2.54	0.39	0.25
% of land cover that is forest	17.2	10.5	23.8	29.0	35.7	10.5
Installed capacity of biomass (MW_th_)	approx. 500	94	2480	1555	1840	280.3
Number of biomass heating & CHP installations	7500	951	32,262	23,431 heating and 18 power plants	33,140 heating and 33 district heating and CHP plants	3414
Area of agricultural land (m ha)	6.91	2.05	2.31	3.85	0.51	1.91
% of land cover that is agricultural	50.2	63.1	65.0	43.9	46.3	80.4
Predominant agricultural land use	Cereal farming and cultivated pasture	Pasture/grassland for livestock	Fruit, vegetables, potatoes, cereals, canola, berries	Olive plantations	Livestock farming and arable crops cultivation	Livestock farming, particularly, dairy cows and sheep

### Germany

The area of SRP in Germany has grown from <500 hectares in 2004 to 5969 hectares in 2014 (DBFZ, 2015). Poplar is by far the most common SRP option and planting peaked in 2010 with around 1400 hectares planted (BMELV, 2012; DBFZ, 2015), falling to 600 hectares in 2015 (von Engelbrechten 2015). The largest area of SRC is in the Brandenburg region with around 50% of the total (Murach et al. 2013). The largest market for SRP poplar is the 5 MW_e_ Märkisches Viertel CHP plant (Vattenfall, 2014).

In 2010, there was a grant available in the Federal State of Saxony paying 30% of the establishment costs of SRC (Faasch and Patenaude 2012). There was also a grant for €1200 for planting SRPs in Saarland in 2012, but this brought about limited planting. (von Engelbrechten 2015). Currently, there is a planting grant for SRC under the Gemeinschaftsaufgabe Agrarstruktur und Küstenschutz (GAK) or Joint Task Agricultural Structures and Coastal Protection Framework Plan (FNR, 2015). This is not a national scheme and only five of the 15 German regions took part in the program: Baden‐Wurttemberg, Brandenburg ‐Berlin, Mecklenburg‐Western Pomerania, North Rhine‐Westphalia, and Thuringia.

### Republic of Ireland

The Republic of Ireland is 85% dependent on fossil fuel imports costing the nation around €6.5 billion per year (TEAGASC, 2014). Energy crops are viewed as one of the ways that Ireland could help itself meet the target for 12% renewable heat by 2020. Over 900 hectares of SRC willow was planted in Ireland between 2006 and 2013 (DCENR, 2014). The increase in farmer interest was boosted by the introduction of the Bioenergy Scheme in 2007 which covered SRC and Miscanthus establishment costs (DAFM, 2015a). Since 2012, the scheme has been launched annually with narrow application windows (less than 2 months).

**Figure 4:**
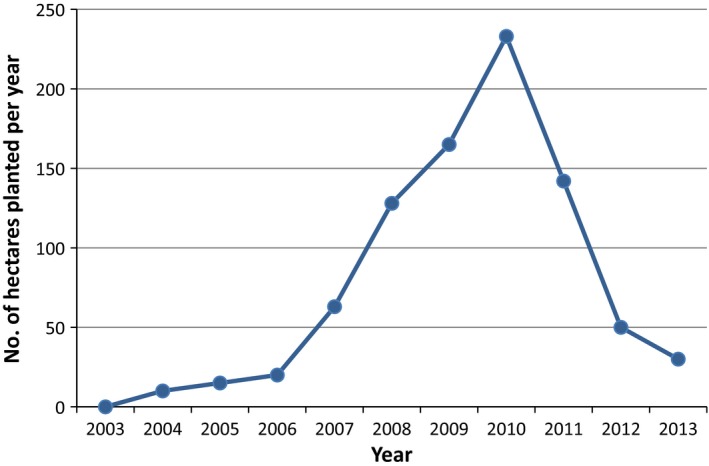
SRC plantings in the Republic of Ireland 2003–2013.

The peak planting year was 2010 when over 200 hectares were planted (see Fig. 4). Planting levels have fallen rapidly since. The largest market for energy crops in Ireland is co‐firing with peat in the Edenderry plant (Bord na Móna, 2015). The main reason for the fall in planting of SRC seems to be the bad press associated with energy crops as a result of the lack of markets for Miscanthus (Independent, 2013; Irish Examiner, 2013). The majority of Miscanthus growers were located in County Cork and County Limerick, too far away from Edenderry site in County Offaly to make it a viable market. This coupled with the low price offered for the fuel led the majority of Miscanthus growers to grub out their crops (Irish Times, 2013).

The Bioenergy Scheme in 2015 now covers just SRC willow with the maximum area that can be planted, increased substantially; but, the maximum grant and percentage of eligible costs covered, both reduced (DAFM, 2015a). It is expected that a Renewable Heat Incentive (RHI) scheme will be introduced in 2016 (DCENR, 2014) which could see an increase in SRC willow planting.

### Poland

Poland is the largest coal producer in EU and its dependency on fossil fuels imports is among the lowest in the EU (Wisniewski and Oniszk‐Popławska 2009). The renewable energy market is dominated by co‐firing biomass with coal in CHP plants. In between 2000 and 2006, regional environmental funds supported local initiatives for SRP introduction, but that resulted in limited planting. Establishment grants at national level for various energy crops (SRC willow and poplar, Miscanthus and *Sida hermaphrodita*) were introduced in 2007–2008 and supported 1300 ha of planting until the scheme was withdrawn in 2009 (Szymańska and Chodkowska‐Miszczuk 2011). Despite the absence of further support, the planted area of energy crops continued to grow from 6193 ha in 2010 to 11,509 ha in 2013, with SRC willow making up the majority of this area (Aebiom 2015; Gajewski 2015).

In 2005, Poland introduced Tradable Green Certificates (TGC) to support renewable electricity production (Heinzel and Winkler 2010). This led to a surge in biomass co‐firing with coal as well as dedicated 100%‐fuelled biomass systems. By 2012, co‐firing alone had increased the biomass consumption to 12 M tons (9.5 TWH electricity generated) at 50 plants. However, a fall in the price of TGCs from the peak of around €62/MWH (275 PLN/MWh) in quarter 3 of 2011 to €23/MWH (100 PLN/MWh) in quarter 1 of 2013 resulted in a large decrease in total biomass consumption to around 8.5–9 million tons in 2014–2015 (Polenergia, 2015).

Several energy utilities including MONDI, KGHM SA, Fortum Poland, and PGNiG TERMIKA SA have provided incentives for planting SRPs either through providing support toward planting and maintenance costs or land‐lease arrangements. As an example PGNiG TERMIKA is currently offering farmers €570/ha (2500 PLN/ha) to plant and maintain SRC willow and a guarantee to buy the fuel produced for 17 years or 5 harvests (PGNiG TERMIKA, 2015).

Despite this, the planted area of SRC has seen a slight decrease since 2013. Furthermore, SRC is not explicitly supported under the Polish CAP scheme for 2015–2020 (Pisarek, 2015).

### Spain

Spain has 18.4 million hectares of forest and is the EU's third most wooded country behind Sweden and Finland (Eurostat, 2015). Estimates suggest that there are 500,000 hectares of Eucalyptus and 100,000 hectares of poplar planted (FAO, 2008; Ruiz and Lopez 2010; Isebrands and Richardson 2014). Most of this is planted as single‐stem trees and used for industrial uses such as pulp wood for the paper industry and veneer production (Parra‐López et al. 2015).

There is a great deal of interest in local supplies of woody energy crops from bioenergy project developers and also policymakers seeking to increase production from marginal farmland. Despite this, the area devoted to SRP for biomass production is mainly restricted to experimental plots (Pérez‐Cruzado et al. 2014). There are only around 150–170 hectares of SRP trials in Andalusia but evidence suggest that high yields are achievable (Kauter et al. 2003; Durán Zuazo et al. 2013). The main reason for the lack of commercial planting is the price competition from abundant waste biomass, especially olive trees and forestry residues (Parra‐López et al. 2015).

The production costs of SRPs are much higher and the price paid by power plants is too low to attract more farmers to plant SRPs. Estimates suggest that the production costs for poplar are €20–40/ton while the price paid by the user is €40–55/ton at the farm gate (equivalent to €70/ton for dry chips), green electricity tariffs would therefore need to rise for the power companies to offer farmers a better deal (Carrasco and Sixto 2007). There have never been any establishment grants to support planting of SRPs in Spain.

Ence is Spain's market leader in the production of renewable energy using forest biomass and energy crop. The company currently runs four biomass power stations at Huelva, Navia, Merida, and Pontevedra with a total installed biomass generation capacity of 220 MW (Ence, 2016).

### Sweden

The introduction of a generous planting grant in Sweden led to a mini boom in planting in the mid‐1990s; at its peak, there were 18,000 hectares planted and over 1250 growers with numerous harvesting machines developed (Rosenqvist et al. 2000; Mola‐Yudego and Pelkonen 2008). However, the reduction of compulsory set‐aside from 15% to 10% in 1996/97 brought about a huge slump with planting levels falling from 2000 to 200 hectares in the space of a year with 10–15 cuttings producers leaving the market (Larsson and Lindegaard 2003).

**Figure 5:**
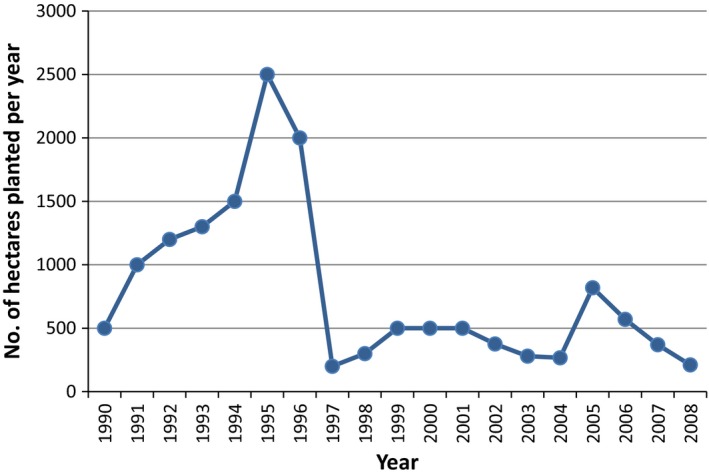
SRC plantings in Sweden 1990–2008 (Larsson 2015).

**Figure 6:**
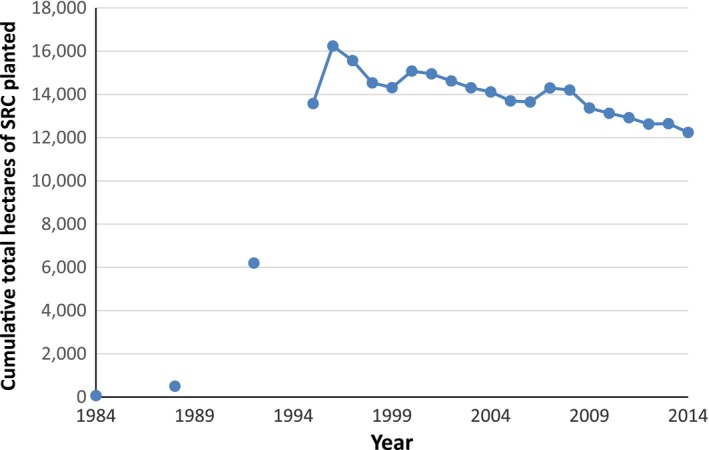
Cumulative SRC area in Sweden 1984–2014 (Sydkraft 1987; Jonsson 1992; Åström and Ramstedt 1993; Swedish Board of Agriculture, 2001, 2003, 2004, 2008, 2011, 2013, 2015; Andersson 2005).

During the years that followed, the Swedish market shrunk due to the removal of crops. Some plantations had been established on poor land hundreds of kilometers from heating plants and were not economical (Helby et al. 2006). Additionally, the price paid to farmers has reduced due to competition from imported biomass and increased incineration of waste. Since the peak planting year of 1996 (see Fig. 5), there has only been two years (2001 and 2008) when a greater area was planted than removed; currently, Sweden has around 12,000 ha of SRC remaining (see Fig. 6) (Swedish Board of Agriculture, 2015).

The main market for SRC is district heating and CHP. In Skåne in 2010, willow contributed only 0.4% of the fuel used in these plants (Nylander 2014). The opening of the 110 MW CHP plant at Örtofta in 2014 and the reopening of the 55 MW Flintrännan heat plant in 2015 could improve the market situation for SRPs (Henriksson 2014).

There is currently an establishment grant worth of €615/ha (5800 SEK/ha) for planting SRC willow.

### United Kingdom

**Figure 7:**
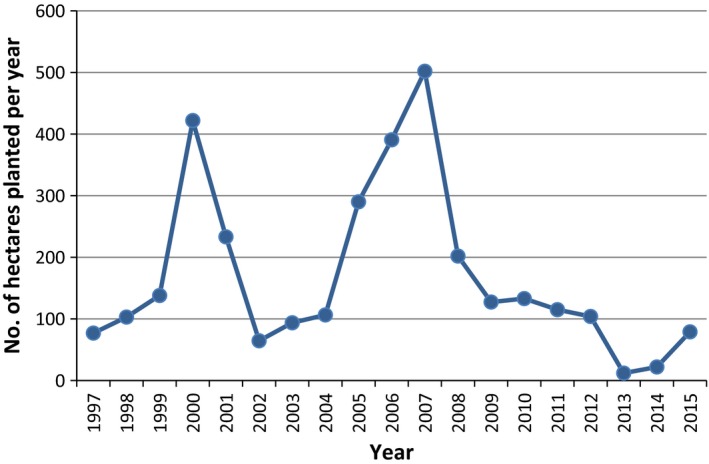
SRC plantings in England 1997–2015.

UK energy crops policy from 1990 to 2015 is assessed in detail by Adams and Lindegaard 2016. Of the four countries making up the UK, only significant amounts of SRC have been planted in England and Northern Ireland. The majority of planting in England is in Yorkshire, East Midlands, and Cumbria. There were two boom and busts experienced in the English SRC industry. The Arbre Energy project was supported by the UK Government's Non Fossil Fuel Obligation (NFFO) and European development funds created a market for around 1500 hectares of SRC willow. The plant was built but never became fully operational and was closed in 2002 (Piterou et al. 2008). Despite the introduction of an establishment grant, farmer confidence was badly affected and planting levels fell from a peak of 422 ha in 2000 to just 65 ha in 2002 (Lindegaard 2013b). The introduction of policy favoring the co‐firing of energy crops with coal led to a gradual increase in planting, peaking at 502 ha in 2007 (see Fig. 7), but this again plummeted due to uncertainty because of the cessation of the Energy Crops Scheme for 18 months, the abandonment of set‐aside, and the sudden increase in cereal prices at this time.

The main market for most SRC growers has been Drax Power Station but they are pulling out from the contract in 2017. There has been a recent small increase in planting due to the completion of the Iggesund Paperboard CHP plant in Workington Cumbria with 50–100 hectares of willow planted for this project in 2015 (Iggesund, 2016).

**Figure 8:**
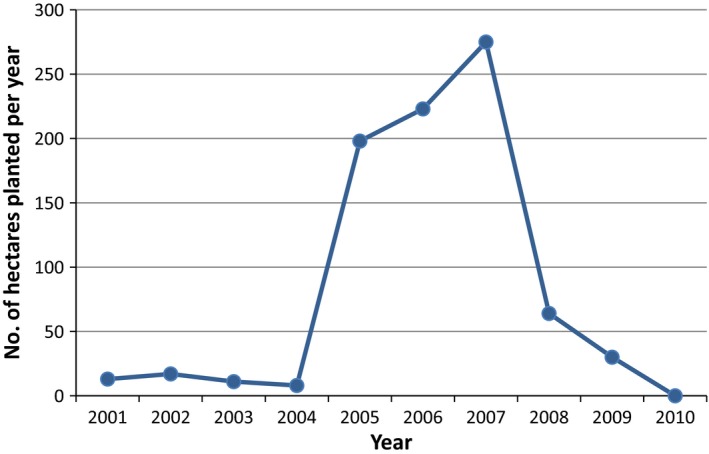
SRC plantings in Northern Ireland 2001–2010.

The majority of SRC planted in Northern Ireland took place between 2005 and 2007 (see Fig. 8) when a scheme called the SRC Challenge Fund was in place (NIDOE, 2016). Most SRC produced in Northern Ireland is used for small scale heat supply (Farming Futures, 2015). A Renewable Heat Incentive (RHI) covering England, Wales, and Scotland was introduced in 2011 (DECC, 2011). This provided a role for some SRC that was already planted but did not lead to significant new plantings. The Northern Ireland RHI which was introduced in 2014 similarly enabled markets for existing plantings; this scheme closed to new applicants in February 2016 (NI Direct, 2016).

### Comparative summary of EU SRP establishment schemes

A comparative summary of the different SRP establishment schemes from the different EU countries is provided in Table 2.

**Table 2:** Summary of SRP establishment grants offered in five European countries

Country		Name of scheme	Type (N = national, R = regional)	Years running	Crops covered	Grant value€/ha	% of total eligible costs	Min. area (ha)	Max. area (ha)	Min. no. of plants/ha	Min. length of plant‐ation lifetime	Notes	Source
Germany			R	2008–2013	SRC poplar, willow, Robinia		30					Saxony only	Faasch and Patenaude (2012)
			R	2015–2018	SRC poplar, willow, Robinia	1200	40	3.75	10	3000	12	5 German regions	FNR (2015)
Ireland		Bioenergy Scheme	N	2007–2014	SRC willow, miscanthus	1300	50	3	30	13,300		900 ha planted	DAFM (2015a)
				2015	SRC willow	1040	40	3	50	13,300			
Poland			N	2007–2008	SRC willow	980	50	/	100			4300 PLN/ha. 1300 ha planted	Szymańska and Chodkowska‐Miszczuk (2011)
					SRC poplar	575	30	/	100			2520 PLN/ha	
Sweden		Omställning 90 (Deregulation ‐90)	N	1991–1996	SRC willow, poplar, hybrid aspen	1065		0.5	n.a.			10,000 SEK/ha. 15,500 ha planted	Blomquist (2006), Mola‐Yudego and Pelkonen (2008)
			N	1997–1998	SRC willow, poplar, hybrid aspen	321			n.a.			approx 3,000 SEK/ha. 242 ha planted.	Mola‐Yudego & Gonza′lez‐Olabarria (2010)
			N	1999	SRC willow, poplar, hybrid aspen	535			n.a.			5000 SEK/ha. 358 ha planted.	Mola‐Yudego & Gonza′lez‐Olabarria (2010)Helby et al. (2006)
		Miljö‐ och landsbygdsprogrammet 2000–2006 (Env and Rur Dev Progr)	N	2000–2006	Willow, poplar, hybrid aspen		40		n.a.			5000 SEK/ha. 1653 ha planted during 2000–2003.	
		Landsbygdsprogram för Sverige år 2007–2013 (Rural Dev Progr)	N	2007–2013	Willow, poplar, hybrid aspen	513	40	1.0 ha willow 0.1 ha poplar, aspen	n.a.			5000 SEK/ha. 1500 ha planted.	Swedish Board of Agriculture (2014).
		Landsbygdsprogram för Sverige 2014–2020 (Rural Dev Program)	N	2014–2020	Willow, poplar, hybrid aspen	615	40	3.45	n.a.	14,500		5800 SEK/ha	Swedish Board of Agriculture (2014)
UK	England	Energy Crops Scheme	N	2000–2007	SRC willow & poplar	1285	/	3	n.a.	n.a.	5	All land types. £1000/ha	Lindegaard (2013a)
						2056	/					Ex forage land only. £1600/ha1815 ha planted.	
				2008–2013	SRC (Willow, Poplar, Ash, Alder, Hazel, Silver Birch, Sycamore, Sweet Chestnut and Lime)	/	50	3	n.a.	n.a.	5	Ca. 900 ha planted	
	Northern Ireland	SRC Challenge Fund	N	2004‐2006	SRC willow	/	n.a.					Competitive scheme. No minimum grant. 696 ha planted.	NIA (2008)

### Current SRP market situation

In most of these western European countries, the current SRP market is static with very little planting taking place. The current areas of growth for SRP are in Eastern Europe. Large plantations have been planted in Lithuania, Latvia, and Ukraine over the last 3–4 years and this looks set to continue (Rokwood, 2015c).

The recent introduction of short rotation coppice (SRC) as an Ecological Focus Area (EFA) option under the current CAP so called “greening” measures was envisaged as possibly having some impact on planting levels (Andersons, 2015 Lindegaard 2013b; Larsson and Henriksson 2015).

Any farm in the EU28 with more than 15 hectares of arable land must have 5% of the land in EFAs. This could rise to 7% after 2017. EFA measures can be thought of as land set aside for environmental benefits. The long list of EFA measures proposed by the European Commission (EC) to member states included SRC. This has been adopted by Germany, Ireland, Poland, and Sweden. In the UK, agriculture is a devolved matter and individual countries make the decisions. SRC has been adopted as an EFA measure in Wales and Northern Ireland, but not in England or Scotland. Neither are likely to have much effect on planting levels although both regions are dominated by grassland (<5% arable).

Each EFA measure is given an EFA weighting that is used to transform the lengths/areas of the EFA measures into equivalent land areas depending on how environmentally friendly they are deemed to be. Where SRC has been included as an EFA measure, it has been assigned one of the lowest weightings (0.3) because it is considered to have a relatively low ecological benefit per unit area. This assessment seems to disagree with numerous studies that suggest that SRC can provide significant biodiversity benefits and ecosystem services (Sage et al. 2006; Rowe et al. 2009, 2011, 2013; Nisbet et al. 2011; Environment Agency, 2015). The low weighting compared to other options (see Tables 3 and 4) discriminates against SRC as it requires as much as 2–7 times the amount of land to be taken up compared to other measures. The weighting of 0.7 awarded to nitrogen‐fixing crops would suggest that these crops are deemed to be more environmentally friendly than SRC. Originally, the European Commission had also proposed a weighting of 0.3 for these crops but this was increased following strong lobbying from the European Parliament (Hart et al. 2016).

**Table 3:** Ecological Focus Area (EFA) options and their weightings (DARD 2014; The Scottish Government 2015, Welsh Government 2015)

Measure	Weighting
Fallow land	1.0
Hedges/Wooded strips	2.0
Buffer strips	1.5
Catch crops or green cover	0.3
Nitrogen fixing crops	0.7
Field margins	1.5
Ditches	2.0
Traditional stone walls	1.0
Archaeological features	1.0
Earth banks	1.0
Agroforestry	1.0
Short rotation coppice	0.3
Afforested areas	1.0

**Table 4:** Tree species permitted to be grown as SRC and their management practices in the EFA options for six European countries (DAFM, 2015b; Hart 2015)

Species covered	Germany	Ireland	Poland	Sweden	UK (Wales)	UK (Northern Ireland)
*Salix spp*	✓		✓	✓	✓	
*Populus spp*	✓	✓	✓		✓	
*Alnus spp*	✓	✓			✓	
*Betula pendula*	✓	✓	✓		✓	
*Fraxinus excelsior*	✓	✓			✓	
*Acer spp*		✓			✓	
*Quercus spp*	✓	✓			✓	
*Tilia spp*		✓			✓	
*Castanea sativa*		✓			✓	
*Corylus spp*		✓			✓	
Max harvest cycle (years)	6	30	3	10	20	5
Mineral fertilizers	Not allowed	Not allowed	Allowed with limits	Only the first year	Not allowed	
Plant protection products	Not allowed	Not allowed	Not allowed	Only the first year	No use of plant protection products, except for spot treatment of invasive non‐native species within the first 2 years of planting	Allowed until end of year 2

## Summary Outcomes of the PESTLE and SWOT Analysis

**Figure 9:**
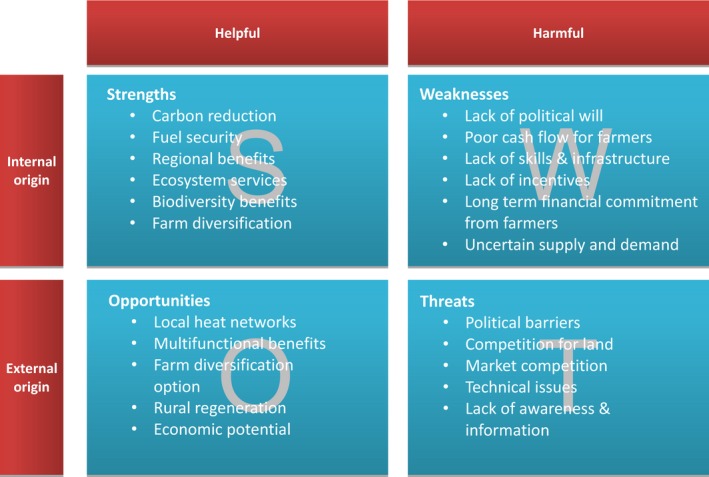
Main outcomes from the SWOT and PESTLE analysis performed for the SRP market.

Findings from the PESTLE analysis have been merged into the SWOT analysis which is described in a Rokwood publication (Rokwood, 2014a); hence, only summarized results are presented here. Developing and prioritizing a combined SWOT for all regions was challenging due to the differing characteristics, circumstances, and priorities for each region but a summary of the SWOT outputs by theme is shown in Fig. 9. Table 5 presents the identified strengths, weaknesses, opportunities, and threats.

**Table 5:** Strengths, weaknesses, opportunities, and threats identified for the SRP market in each region (N.B. these apply to all regions unless otherwise stated)

Strengths	Weaknesses
Carbon Reduction Reduced CO_2_ emissions and greenhouse gasses Implementation of international commitments to reduce emissions e.g. EU 2020 Increasing the role of renewable energy in national and regional energy policies. Building Regulations – RES requirements and Carbon compliance targets. Fuel Security Stable energy supply to meet demand Advances in technical development make SRP a good long‐term fuel option Increase in energy coming from renewable sources which most governments support. Regional Benefits Provides sustainable rural development Flexible in scale to fit a particular area Employment potential for the local area Suitable climate for growing SRP (Sweden/Ireland/UK in particular). Economic Stimulates the national economy, particularly the agriculture sector In the long term will lead to cheaper heating Some countries have grants/funding from governments to support SRP (see section Short Rotation Plantations (SRP) policy review) Existing expertise – willow breeding (UK), whole supply chain (Sweden). Biodiversity Promotes flora and fauna better than traditional mono‐crops Willow in particular supports invertebrate species Reduces soil erosion. Added Benefits Using sludge as fertilizer Multifunctional crop Water treatment option Provides a natural windbreak Acts as a flood defence.	Land Limited land availability Not all land is suitable for SRP Established traditions of land use are difficult to change Protection of the landscape is an issue in some areas. Lack of Political Will Certain agriculture rules and regulations may impede the process Lack of joined up thinking from policymakers for how SRP can achieve multiple goals Lack of subsidies/grants to establish industry (in some cases) Focus on other alternative energy sources, e.g., wind, biogas. Lack of support Lack of awareness by most of society Skepticism of the technology and opposition to change Resistance to change by producers, including peer pressure to maintain existing practices Minimal lobbying for change. Lack of Skills and Infrastructure Need to further develop the technical infrastructure, e.g., combustion systems Harvesting infrastructure is limited (UK, Ireland and Spain) Lack of profitable specialized machinery for SRP (Germany, Poland and Sweden) Lack of training for best practice in both agricultural and business Lack of working examples demonstrating the possibilities and as a way of knowledge sharing. SRP Market Lack of established market for SRP Higher price compared to some other fuels Lack of collaboration between relevant stakeholders to develop market All of the above creates uncertainty for potential investors. High Costs Establishment – long‐term investment is required Poor cashflow for farmers, does not provide a good return in the short term Transport – potential long distances involved Combustion – new area requiring investment Grid connections are expensive where they do not exist. Operational Issues Storage and drying of high moisture content woodchips No guarantee that heating plants will accept SRP Drainage issues of the land while growing Transprt logistics need to be developed.
Opportunities	Threats
Political Potential to make required legal changes, e.g., making SRP a subsector of forestry Possibility of being included in beneficial CAP policies Taxes on fossil fuels could further advance economic advantage Positive environmental impact such as carbon reduction is good for national/council targets. Regional Good for rural areas where gas use is low and alternative heating sources are expensive Potential to reduce fuel poverty (UK) Use marginal land that is not currently being utilized Opportunity to engage local government in energy matters Reduce logistical issues by promoting local use Create more local jobs. Economic Potential to provide good value heating in the long term Possibility of government funding Increasing price of fossil fuel internationally makes biomass more attractive International trading possibilities including “high grade” SRP. Promotion of SRP Need to challenge negative public opinion Promotion of the use of pellets/wood chips to stimulate demand Target young farmers and farm sectors most likely to adopt SRPs. Possible Benefits Sewage water for irrigation Remediation of brown‐field sites Development of ecosystems. Technical Improvements Better quality due to improved SRP varieties and harvesting techniques Possibility of linking to heat networks and CHP.	Political Barriers Agricultural reform may prove negative New emissions criteria targeting NOx emissions could pose a problem for nitrogen rich willow Tax issues for energy crops Conservation laws and regulations (Germany and Sweden) Bureaucracy creates complexity Isolated local authorities lack leadership Inconsistent policy and regular changes leading to uncertainty. Technical Issues High levels of particulate matter (air pollution) possible in urban areas due to large‐scale domestic biomass Need for improved air pollution mitigation measures, e.g., filtering technology Lack of a plan to change existing power generation (locally and nationally) to biomass Tree diseases resulting in a glut of wood fuel. Economic Difficult to compete with sources of waste biomass Possible reduction in government funding Tenant farmers have insufficient funds to invest Competition from other fuels, e.g., gas, coal, oil, kerosene Immature market and limited current development Subsidies have tended to promote energy generation, not feedstock supply. Market Competion for land from crops and other uses (land use change) No competitive advantage over imported biomass Expansion of gas infrastructure Competition from other renewable energy sources High food prices lead to an unwillingness to use land for energy crops In some countries Miscanthus is a more popular energy crop with farmers (UK) Changing policy leads to market uncertainty and reduces investor confidence. Limited R&D Future funding of R&D Bioenergy funding often more focused on technical solutions overlooking feedstock supply.

### Strengths and weaknesses

The hypothesis used in this study is that the positive benefits outweigh possible negative impacts of SRP, which is supported by several studies (Rowe et al. 2009; Langeveld et al. 2012; Adams and Lindegaard 2016). Key environmental benefits include increased carbon sequestration, reduced GHG emissions, reduced soil erosion, and groundwater nitrate and surface runoff. Also, SRP can be used in phytoremediation of contaminated land and can lead to an increase in biodiversity. Furthermore, most of the unfavorable impacts can be managed and mitigated. Land use can be restricted to suitable sites such as marginal land, contaminated land, buffer strips, and lower quality agricultural areas (ADAS, 2008; Styles et al. 2016). For hydrology, guidelines on catchment management can be enforced to ensure detrimental effects do not occur to hydrological resources (Rowe et al. 2009).

Fuel security is a key strength of SRP due to local production and reduced imports which highlights some regional benefits. Local supply is good for employment and farm diversification as it allows farmers an alternative income stream from a different enterprise. Despite these strengths, SRP currently offers a poor cash flow for farmers and there is a lack of political will to support SRP which contributes to uncertainty in supply and demand. A lack of incentives has limited uptake of SRP which is also compounded by a lack of skills and infrastructure. SRP requires significant land take and a long‐term contractual commitment with the landowner which is recognized as a weakness. A general lack of public awareness of the industry, the supply chain and end‐user benefits also featured highly as a weakness.

A number of factors were found to be specific to each country or region due to variations in market advancement, existing national/local policies, and the local characteristics of the area. For example, the possibility of using SRPs to assist flood mitigation was included as a strength by the UK region, which reflects the high incidents of flooding experienced in the South West of England. The UK, Irish, and Spanish regions noted weaknesses in the lack of harvesting infrastructure and supply chain logistics, while the Swedish, Polish, and German regions identified a lack of profitable specialized machinery for SRP and lack of technological development to address this.

### Opportunities and threats

While the results varied across the regions, there was a noted dominance of political and economic issues that could either be viewed as opportunities or threats depending on policymakers' decisions. Common Agricultural Policy (CAP) reform and the role of SRC in Ecological Focus Areas (EFAs), government national policy, and the extent to which SRP is prioritized and supported, and EU/national targets for renewables and emission reductions – all featured highly in this respect, with most being viewed as opportunities. This made clear that with the appropriate political encouragement, backed up by the right economic incentives, the SRP market could be kick‐started to ultimately compete on an equal footing with other feedstocks in the sustainable heat market. The wider issue of increasing fossil fuel costs was noted by all regions as a significant opportunity in this respect.

Local heat networks offer a substantial opportunity for SRP as it builds on the strengths of fuel security and regional benefits which benefits rural regeneration. Diversification of agriculture and farming offers multifunctional benefits such as economic potential and environmental enhancement. A key opportunity for SRP is to combine the energy production with other opportunities to improve ecosystems services such as flood mitigation, water treatment, and reduced runoff.

Common threats included a lack of local markets, with the more advanced regions also highlighting the risk of local markets being affected by an increased import of cheap biomass fuel and the low prices attracted by the biomass power sector. Regulations around landscape protection and nature conservation were viewed as threats by the German and Swedish regions. Market competition and technical issues were identified as threats in all regions due to alternative renewable energy technologies, competition for land, and problems such as air pollution, combustion efficiency, cheap imports, and limited species optimization for SRP. Awareness of SRP and relevant information in the public domain is a threat to obtaining future support for market development.

Potential negative aspects of SRP cultivation warrant further assessment and consideration before making policy recommendations to support SRP. It is important that SRP is grown in appropriate locations as competition for land and concerns over land use change is a crucial issue (EEA 2006; ADAS, 2008; DECC, 2012), for example, cultivating SRP on permanent unimproved grassland can lead to soil carbon loss (Rowe et al. 2009). Indirect land use change (ILUC) and “food versus fuel” is an ongoing debate which is a limitation restricting the development of SRPs in many countries with limited land availability (Berndes et al. 2011; Ecofys, 2015). Some trees managed as SRPs are planted as monocultures and exotic species such as Eucalyptus provide few biodiversity benefits. There are also concerns around water availability due to considerable water requirements, landscape change due to visual impacts, and potential for deep roots to affect archaeological remains (Finch et al. 2009).

In summary, the SWOT analysis highlighted the key benefits and risks associated with further support for SRPs. The key barriers are assessed in section Policy recommendations along with policy recommendations to increase the uptake of SRP.

### Policy Recommendations

In this section, the results of the regional policy briefs are amalgamated and assessed as there was a high degree of alignment in the issues identified by the countries. Six broad requirements emerged, with each identified by more than one region as an area where appropriate policy change was required. A summary of the problems and potential options toward a solution are summarized in Table 6 with a more detailed description provided in the subsections below.

**Table 6:** Summary of the policy recommendations, problems identified and potential options toward a solution

The Problem	Toward a Solution
Better dissemination of information regarding the benefits of SRP	
Need to educate relevant groups about the benefits of SRP	Provide courses, disseminate information via literature and workshops/events.
A coherent body of evidence of benefits of SRP in one place is currently lacking.	Increase weighting factor of SRC to 1.0 in Ecological Focus Areas (EFA).
	Conduct a full evidence‐based review of SRP including a cost benefit analysis.
	Further research into the multifunctional benefits of SRP to society.
Increased financial support to foster the SRP market	
Need for greater financial support to grow the nascent SRP market.	Additional funding from regional or national government to kick‐start industry.
	Regional establishment grants, interim payments during the establishment period, interest‐free loans and subsidy payments.
Developing the supply chain at the local level	
Lack of local supply chains is a barrier to the uptake of SRP. This leaves growers isolated and lacking adequate infrastructure.	Provide subsidies in areas where the SRP market is able to grow. Grants for crucial infrastructure could be made available.
Often supply is not linked to end‐user demand leading to imbalances.	Establish pilot projects that connect growers to end users. Create a strong demand for biomass through taxes and perception.
Improved clarity regarding SRP funding and land use	
Broadening definitions to include SRP in environmental stewardship, biomass, forestry, and agricultural support schemes.	Improve legislation so that SRP can be incorporated into land sector support schemes to increase competitiveness.
Issues over the suitability of different land use for SRP, e.g., forestry or agricultural.	Improved classifications and clarifications of land use so that farmers can make informed decisions about SRP.
More research and development in SRP leading to better resources	
Continued R&D on specific aspects of SRP cultivation to increase commercial viability.	Appropriate funding for research programs in EU countries.
	Increase pilot projects and field trials and work closely with policymakers, industry, and researchers to maximize value of R&D.
Formation of a policy development group	
Lack of lobby groups supporting SRP and limited policy development has hindered development.	Formation of lobby groups to improve the way that Government deals with energy crops policy.
More political support is required as policy‐making often falls between different Government departments.	Potentially push for an interdepartmental body for energy policy to ensure that different Department's objectives are aligned.

### Better dissemination of information regarding the benefits of SRP

#### The Problem

All of the regions identified the need to educate relevant groups about the benefits of SRP, particularly farmers and policymakers. The UK region identified a lack of knowledge regarding the potential positive social and economic impacts of SRP such as reducing fuel poverty by providing cheaper fuel and job creation from a new industry. Spain recognized the potential of SRP to provide cheaper fuel to rural communities with no access to mains gas. The Swedish region also highlighted a lack of awareness of SRP's potential to provide energy security to the nations in which it is grown.

Despite there being multiple environmental benefits of SRP, a coherent body of evidence in one place is currently lacking. This undermines efforts to assess these benefits in a holistic way. The German and Swedish regions state that the positive ecological effects of SRP need a much greater focus. This would include the ability of SRP to: regulate groundwater levels, clean wastewater, prevent ground erosion, and increase biodiversity (Langeveld et al. 2012). These benefits are significant when compared to alternative energy crops such as maize (Farnworth and Melchett 2015), however, they frequently go unnoticed. This is in part due to a lack of practical examples and evidence (in particular, as a potential source of flood mitigation) of the value of these multifunctional benefits.

The lack of clarity on the environmental benefits of SRP has been pinpointed by the Swedish region as a large contributory factor to the halting of their progression in Sweden, a country which originally led the way in developing the production of SRP. The very low weighting for SRC as an EFA option under the CAP 2015–2020 means that if farmers choose to grow SRC, the proportion of land they must turn over to EFAs increases (Hart 2015). This sends the signal that SRC is less environmentally friendly than other crops, such as peas and beans which have a higher weighting. This has played a part in the slowing of development of SRC in Sweden and could have similar implications in other EU countries (Hart et al. 2016). For instance, in Germany, the area of SRC as an EFA measure in 2015 was just 2200 ha out of a total area of 1,367,400 ha. In contrast, nitrogen‐fixing crops covered an area of 161,800 ha and catch crops and cover crops covered an area of 930,200 ha.

The Spanish region states that Spain suffers from a lack of knowledge regarding heat production in general and of SRP in particular (Parra‐López et al. 2015). This is at multiple levels, from farmers all the way to the general public. Similarly, the Irish region identified a lack of understanding of the sector by prospective growers.

Results from the Polish workshop suggest the lack of knowledge on SRP is also widespread; even those in charge of making energy and heat production policies are largely unaware of its existence. The lack of locally available infrastructure has resulted in some producers trying to adapt machinery to the biomass process or to build their own systems, resulting in an inefficient and suboptimal production process. This is not a conducive environment to promoting the benefits of biomass production.

#### Toward a solution

The methods suggested for better targeting of information to relevant stakeholders varied, with various regions suggesting different strategies. The proposed solution in the UK and Germany is to conduct a full evidence‐based review of SRP including a thorough cost benefit analysis. An up‐to‐date evidence base would facilitate a process of clarifying opportunities which in turn could be used to raise awareness of the range of additional benefits around biodiversity and ecosystem services that SRP can provide (Lindegaard 2013a,b) and potentially lead to greater funding through environmental schemes. The Swedish region found that more research on SRPs in general is required, and in particular, on the potential economic value of SRP multifunctionality to society. Both researchers and industry should push for funding for well‐documented research and experimental field trials to show the benefits to industry stakeholders and policymakers.

If firm conclusions are drawn from this research about the strong environmental benefits of SRP, then it is important that a review is made of the weighting given to SRC in the EFA guidance. Increasing the weighting factor of SRC to 1.0 would send an important signal to farmers that it is an environmentally beneficial crop that is worth cultivating. This might encourage countries like England, who have not adopted SRC as an EFA option to reconsider.

Spain suggested a wider range of options to tackle this problem through the provision of information to all stakeholders (politicians, energy generators, farmers, and the general population) through courses, networking, campaigns, and technical visits. This would be done at all policy levels, from national to local. As with Germany, the hope is that more informed policymakers would increase the levels of funding available. Ireland recognizes the important need of countering not only the lack of knowledge in some cases but also some of the popular misconceptions regarding SRP. They advocate a program of training and education but also focusing on farmers involving the development of factsheets, workshops, and seminars. This would cover the whole wood fuel production process including: which varieties are suitable to the local climate and soil conditions, the management techniques required, harvesting and drying processes, and applications for end use.

Poland suggested that the best way to increase knowledge of the SRP process was to provide working examples of the stages in the biomass production chain. For instance, EU or national funding could be sourced in order to build biomass heat production facilities in local communities which would serve as a prototype for those hoping to invest in similar facilities. It is also suggested that building contacts with institutes dealing in the biomass market abroad might help to disseminate the necessary knowledge.

### Increased financial support to foster the SRP market

#### The Problem

The regions identified a need for greater financial support as an issue that needs to be addressed in order to grow the SRP market. The UK region recognizes the need to lower the investment risk for SRP growers. This is largely due to the view that SRP is a high‐risk, long‐term commitment that most farmers are unwilling to undertake. Sweden also recognizes this issue; it cites the example of *Salix* grown as SRC which needs an investment time of 20–25 years before it turns a profit, with the first 8–10 years likely to result in negative cash flow. Financial support may be necessary in order to support farmers in taking the economic risk of turning land over to SRP production. The German region also highlighted the lack of funding for SRP growers; this is particularly significant as currently funding is available for other, more traditional crops making them more attractive than SRP. Ireland, similarly, noted a funding discrepancy, as relatively generous grants are available for forestry but this is not currently defined as including SRP. The result is similar to Germany whereby farmers are unlikely to grow SRP without a subsidy when funding is available for other crops. Spain does not specifically focus on financial incentives as the subject of any of their policies. However, they do include additional funding as a necessary part of the solution in broad terms regarding all of their other policies.

In Poland, extra funding was also observed to be necessary, in this case not to compete with other crops but with cheaper fossil fuel energy production. In Poland, the energy market is dominated by cheap coal and currently the same amount of energy can be produced from a much smaller tonnage of coal at a substantially lower price. Thus, if biomass is to get a foot in the door, then financial incentives are critical.

#### Toward a solution

All of the regions are in broad agreement that additional funding should come from regional or national government in addition to other funding sources. The UK recommends that government at both the national and local levels work together to improve finance, with the local element creating opportunities for targeting of schemes in appropriate areas that create the most benefit. The complete package of support could include a variety of attractive options such as crop regional establishment grants, interim payments during the establishment period, interest‐free loans, and subsidy payments. The latter two options would be in the event of the SRP planting aiding in flood defence or water treatment. Germany envisages the motivation coming primarily from local government, through engagement with national farmers' unions to make use of existing CAP funding infrastructure, specifically, allowances for greening (Hart 2015). They argue that by increasing the EFA weighting factor for SRP, the level of funding available would increase and make these crops a more attractive financial proposition.

Ireland proposes two funding options, one possibility would be to make use of the current grant that supports forestry by extending it to include SRP thereby allowing SRP to compete financially for land use. The Irish partners also called for a renewable heat incentive (RHI) which would lead to a market pull for energy crops (as a result of the country's low indigenous woodland cover). It is hoped that such a measure could help Ireland in achieving its 2016 RES‐H targets; a target which if not achieved may have large financial implications (TEAGASC, 2014).

In Poland, there is precedence of EU funding being used to realize the development of community projects. At present, however, funding is prioritized toward projects focused on other renewable energy sources, such as wind and solar. The biomass sector must therefore present a strong case for accessing this support mechanism.

### Developing the supply chain at the local level

#### The Problem

Three of the regions, the UK, Germany, and Spain identified the lack of local supply chains as a barrier to the uptake of SRP. Poland and Sweden highlighted, in particular, that a lack of retail market for SRP was likely to hinder its success. They all saw the development of such supply chains via their policies to be a potential opportunity.

There are only two instances of established supply chains in the UK, serving the Iggesund Paperboard CHP plant and Drax Power Station (Iggesund, 2016, REGRO, 2016). Outside these regions, the supply chain is struggling to develop as growers are relatively isolated and infrastructure and specialized machinery required is not sufficient for economies of scale. The investment risk means there is still little incentive to invest in the necessary infrastructure. The workshop identified a pressing need for locally available infrastructure, so that smaller growers can join the SRP market and produce the properly prepared wood fuel which will allow access to the lucrative domestic energy market.

The German workshop recognized the importance of developing the supply chain as a possible means of regional development, citing that some parts of the country such as the Saxony‐Anhalt region are quite economically deprived.

The Spanish region identified a barrier to SRP – overcapacity in installed power due to the large growth over the last decade of wind farms and combined cycle gas turbine (CCGT) plants (STORE, 2013). This is likely to mean a very competitive electricity market and low prices paid to SRP growers. By focusing on local district heating systems, this barrier to SRP uptake could be overcome. They also see local biomass networks as a way to increase energy security and independence.

In Sweden, there is quite a well‐developed production and supply chain. However, the lack of the final link in the chain, a home market to support their biomass production, is threatening to cripple the industry. Poland has a similar and serious lack of a retail market for any biomass produced as the country's infrastructure is set up to use coal, from large coal‐fired power stations to the coal‐burnings stoves in individual houses.

#### Toward a solution

The broad solution from each of the regions is that subsidies are required to stabilize the market and that this should be focused in specific areas where the SRP market is able to grow. The solution to this issue proposed by the UK is to establish regional pilot projects that connect growers to end users. This would involve setting up one or more biomass heating, CHP, or district heating systems. The Rokwood case studies showcased other projects such as the Beuchte Bioenergy Village in Germany (a district heating scheme linking 65 households) that could be replicated elsewhere (Rokwood, 2015c). It is suggested that the required funding could come from the Rural Development Program, with targeted loans and leasing arrangements to help growers establish SRP (Adams and Lindegaard 2016).

In order to facilitate the move to biomass district heating, the Spanish region propose that grants and subsidies should be more favorable to locally sourced biomass including SRPs because of their lower lifecycle GHG emissions compared to imported biomass (Adams et al. 2013).

The expansion of SRP is already happening in specific parts of Germany such as the Achental region where the Biomassehof project is connecting producers and consumers of biomass locally (SRCPlus, 2014; Rokwood, 2015c). It is plausible that this could extend to other parts of the Saxony‐Anhalt region, and that the creation of local supply chains could be an opportunity to increase the economic prosperity of struggling regions. As such, the German policy proposal advocates extending current networks, especially in rural areas with economic difficulties (Rokwood, 2015d).

There is a belief from Polish stakeholders that focusing on creating a strong demand for biomass will help the rest of the chain to fall into place. Legal and financial measures will be needed in order to create this buyers' market, and this would entail external funding from the state or the EU. The current 2014–2020 Development Strategy (which aims to build the capacity in each community for renewable energy production) could offer an opportunity to build on this aim (Rakowski 2015).

### Improved clarity regarding SRP funding and land use

#### The problem

Three of the regions recognized opportunities for improved clarity that would support the expansion of SRP. This ambiguity exists in different areas; for Ireland, it affects funding, in Spain and Germany, there are land use issues.

Ireland notes there is a need to examine the specific definitions of biomass currently used in the country. The region advocates the possibility of broadening these definitions in order to bring additional funding streams to different varieties of SRP. This would mean including other crops in the existing bioenergy schemes as opposed to just willow and previously Miscanthus. Additionally, there is also a pressing need to identify the lifetime gap between grants which support forestry and those which support SRP to determine new potential support structure.

In Spain, there are issues with regard to which areas of land are suitable for SRP: agricultural or forest. This confusion partly arises due to the different SRP species which can be planted. There are similar land use uncertainties in Germany. The legal status of the land used for SRP production has caused a great deal of confusion as it was unclear whether farmland with SRP remained farmland or became woodland after a certain period of cultivation. An amendment to the German Federal Forests Act in 2011 deemed that SRC plantations are not considered to be forests as long as the harvest is performed within 20 years (BWaldG, 2011). This should have enabled German farmers to claim farming subsidies but has not happened in practice. An additional problem is unused land which SRP could help bring back into use but which is currently being underexploited.

#### Toward a solution

The regions are in broad agreement that policymakers need to do more to foster SRP through improved classifications and clarifications of land use so that farmers can make informed decisions about SRP.

In Ireland, this would be the responsibility of the relevant government departments and state agencies to re‐examine the current Bioenergy Scheme. They would need to look at the support offered to forestry growers and develop a plan to address the disparity between this and what is offered to SRP growers. Ireland envisages that the policy development group (discussed in section Formation of a policy development group) would liaise with the relevant government departments to re‐evaluate the definition of energy crops in a positive way.

Spain, very clearly advocates the need for new national legislation as a way to address this issue. This is required to redress the financial disparity between SRP and forestry in terms of financial support as currently the energy crops industry cannot compete with forestry. By legislating for increased subsidies for SRP, interest and participation in growing energy crops is expected to increase which should help Spain reach their RES‐H target. The benefit of re‐classifying energy crops and the widening of the Bioenergy Scheme in Ireland, would allow for the development and promotion of native species and royalty‐free species into the market. Spain is also looking for clearer criteria on land use with regard to agricultural and forest land and specific parameter on SRPs in CAP regulation.

Germany proposes a different solution; in recognition of the large amount of fallow, unused land available, they envisage local authorities promoting SRP as a way to bring this land back into use. The potential plots owned by local authorities could be made available to farmers to plant SRPs. In a similar vein to Ireland, Germany sees a role for their national farmers union to advocate for SRP, though current organizations are seen as sufficient without the need for a new lobby group as suggested by Ireland.

### More research and development in SRP leading to better resources

#### The problem

Three of the regions, Spain, Sweden, and to a lesser extent the UK, note the importance of continued research on SRP as part of developing better resources. The Spanish region identified the need for research to identify SRP species and varieties more adapted to the warmer climate as well as suitable farming methods and technologies.

The UK recognizes that in addition to consolidating the current body of evidence into a consistent format to share more widely (as discussed in section Better dissemination of information regarding the benefits of SRP), further research also needs to be undertaken to ensure benefits and drawbacks are understood in terms of a holistic whole. There is also support for this in the Swedish region, who suggest that more research is needed into the multifunctional benefits and ecosystem services provided by SRP. Without furthering the knowledge base, incentives for SRP production are not being implemented and opportunities are therefore not being realized. Through consolidating existing evidence, research gaps would be identified which in turn would help prioritize future actions and priorities for SRP.

#### Toward a solution

The issue of increased research will necessarily work in tandem with other policy goals, particularly the key point of wider dissemination of information outlined in section Better dissemination of information regarding the benefits of SRP. Achieving this goal will rely on necessary funding for research programs and working closely with relevant institutions.

Spain recommends a range of policies to help solve this issue. One suggestion is closer working with universities and research institutions to develop new species and machinery to ensure better yields. Another component of this is to conduct further research into methods that use less fertilizer and weed control agents as well as further work examining more efficient use of water and wastewater. In a similar vein to the UK, Spain also suggests starting pilot projects to work on improving the adaptability of SRPs to marginal lands. The Swedish partners are in broad agreement that more field trials are required to demonstrate the environmental benefits of SRP in practice. Such field trials could inform best practice protocols for planting SRPs for environmental benefits and reduce the risk of poorly sited crops that have detrimental effects.

The UK states that identifying and addressing research gaps that increase the overall knowledge and understanding of SRP will in turn trigger constructive and coordinated policy initiatives and incentives. This could work in tandem with the pilot‐based schemes advocated by the UK (see section Developing the supply chain at the local level) to demonstrate possibilities. An example project could involve the use of SRP in flood mitigation in addition to energy supply.

### Formation of a policy development group

#### The problem

The Irish and Polish regions both identified that the lack of lobby groups supporting an increased uptake of SRP was hindering its progress. The Irish region identified the lack of policy development for SRP in Ireland as an issue. They attribute this to the lack of lobbyists in this sector compared to other players in the energy market (oil, gas, electricity, and wind). The need for a cohesive group representing the energy crop industry has been identified by stakeholders.

This view was also presented by Poland, who believed that that marginalization of biomass in legislation and in business decisions was due to a lack of political backing. The coal industry is well established, with all the country's infrastructure set up to serve it and massive lobbying power behind it. Political backing and lobbying will be necessary if biomass is to be recognized in laws of regional and national importance. The Polish region is clear that until local and national authorities begin to be lobbied to include biomass in their development strategies, the achievement of more specific goals will not be possible.

#### Toward a solution

The Irish group believes that support must be provided to help grow a fledgling group who would start to lobby for improvements in the way SRP and energy crops are dealt with by government. An initial goal of this group might be to push for an interdepartmental body for energy policy. This is because there is a lack of continuity in governmental responsibility for biomass with multiple departments having responsibility for different aspects. The Polish region also suggests formation of political support groups to lobby local and national authorities and to make a stand against the large lobbying power of the coal industry in the country.

## Concluding Remarks

The issues that must be tackled in order to ensure the creation of a successful path to market for SRP are numerous and complex, but not insurmountable. However, there is a lack of awareness of the multifaceted benefits of SRP at the level of both farmers and policymakers and thus a coordinated top‐down and bottom‐up approach is needed in order to promote the widespread uptake of SRP. The strategic planting of perennial biomass crops in arable farmland to increase landscape heterogeneity and enhance ecosystem function is recommended to strike a balance between energy and food security (Haughton et al. 2015).

At present, there is a lack of knowledge of SRP as both a feasible crop choice for farmers and as an energy source for heat producers. This is due both to a lack of dissemination of knowledge and the absence of recognition of SRP in governmental policy. SRP is unlikely to be looked on favorably by farmers and producers unless it is afforded the same benefits, subsidies and support that other crops and fuel sources receive from the government. Change in policy is crucial and the support of bureaucrats at the district, regional, and national level is vital for this to be effected. Researchers will also play a part in ensuring there is clear and concrete evidence in the field of the environmental and socioeconomic benefits of SRP. The simultaneous dissemination of this knowledge upwards to policymakers and downwards to producers and farmers is critical in the success of SRP. The formation and development of groups to lobby for the uptake and support of SRP and bioenergy is also of great importance.

The summation of the policy briefs of the six regions of the Rokwood project highlights that there are common obstacles to the wider uptake of SRP across each of the different countries. It also highlights, however, that there are some issues that are unique to one region that are the result of specific circumstances within that country, for example, a structure of governance or the characteristics of existing energy markets. There are similarities in the solutions offered by the regions, but again the variations between them highlight the importance of specific policy changes which are locally relevant.

## Conflict of Interest

None declared.
